# Lateral Approach for Regenerative Treatment of Intrabony Defects Associated With an Edentulous Alveolar Ridge: A Prospective Case Series

**DOI:** 10.1002/cre2.70094

**Published:** 2025-03-02

**Authors:** Filip Hromčík, Adéla Halusková, Lydie Izakovičová Hollá

**Affiliations:** ^1^ Clinic of Stomatology, St. Anne's University Hospital, Faculty of Medicine Masaryk University Brno Czechia

**Keywords:** edentulous ridge, intrabony defect, periodontal surgery, regeneration

## Abstract

**Objectives:**

This case series evaluated the clinical efficacy of the novel “lateral approach” combined with an enamel matrix derivative (EMD) and bone grafting in the regenerative surgical treatment of intrabony defects associated with an edentulous ridge.

**Material and Methods:**

The innovative flap, called the “lateral approach,” is explicitly designed for regeneration of unchallenged isolated intrabony defects associated with *edentulous* alveolar ridges. The flap is defined by a curved vertical incision on the buccal side opposite the treated defect and a sulcular incision on the buccal and defect‐associated sides, promoting uneventful healing and regeneration while minimizing complications.

Seven intrabony defects (one per patient) distal to the lower second molar were treated using the “lateral approach” combined with EMD and grafting with deproteinized bovine bone mineral. The primary outcome was clinical attachment level (CAL) change. As additional parameters, pocket probing depth (PPD) reduction and complication rate were analyzed. All the outcomes were assessed 6 months post‐surgery and compared with the baseline values.

**Results:**

Primary wound healing occurred in 100% of cases, and no complications were reported. At the 6‐month re‐evaluation, the initial median CAL of 6 mm (interquartile range 5–8 mm) was reduced to 3 mm (3–5 mm). The corresponding median PPD was reduced from 6 mm (IQR 6–8 mm) to 4 mm (IQR 3–5 mm). These differences were statistically significant (*p* < 0.05).

**Conclusions:**

The “lateral approach” is a technique for the surgical treatment of intrabony defects associated with the edentulous ridge. Within the limitations of the study, this method seems to be suitable for distal intrabony defects in the lower second molars, which frequently develop after third molar extraction.

## 1 Introduction

Regeneration of the periodontium is the ultimate goal of periodontitis treatment. However, successful regeneration requires addressing space provision, wound stability, undisturbed healing, and availability of specific cell types (Stavropoulos et al. 2022; Nibali et al. 2021; Sanz et al. 2015). This can only be successfully applied to periodontal defects with intrabony patterns of bone resorption (Kyung et al. 2021).

Most research in this field has focused on interdental intrabony defects associated with the papilla, as papillary management is considered a crucial aspect of regeneration. Many different surgical approaches have been proposed, evaluated, and compared to preserve the stability of related soft tissues and provide an appropriate environment for regeneration. Several studies have confirmed that stable soft tissues, specific flap designs, and minimally invasive microsurgical techniques are crucial for periodontal regeneration, irrespective of the biomaterials used (Cortellini and Tonetti 2011; Aslan et al. 2020; Trombelli et al. 2010). On the other hand, some studies did not find significant differences in clinical outcomes between minimally invasive and conventional surgical approaches (Windisch et al. 2022; Schincaglia et al. 2015).

In contrast, very little attention has been paid to defects associated with edentulous ridges. For example, little effort has been made to develop specific flaps for soft tissue management in cases where the papilla is not involved. To date, the widely accepted and unchallenged gold‐standard flap design for such cases, as described by Cortellini and Tonetti (2015), includes a crestal incision right above the treated site, sulcular incision, and, if necessary, release of vertical incision(s) and reflection of both buccal and oral flaps. The most common complications of this approach are membrane exposure, graft contamination, and marginal flap dehiscence, leading to improper healing and insufficient results (Machtei 2001; Cortellini et al. 1990; De Sanctis et al. 1996). In cases treated with membranes, the occurrence of such complications ranges from 50% to 100% (Cortellini and Tonetti 2015; Sanz et al. 2004; Jepsen et al. 2023).

**Figure 1:**
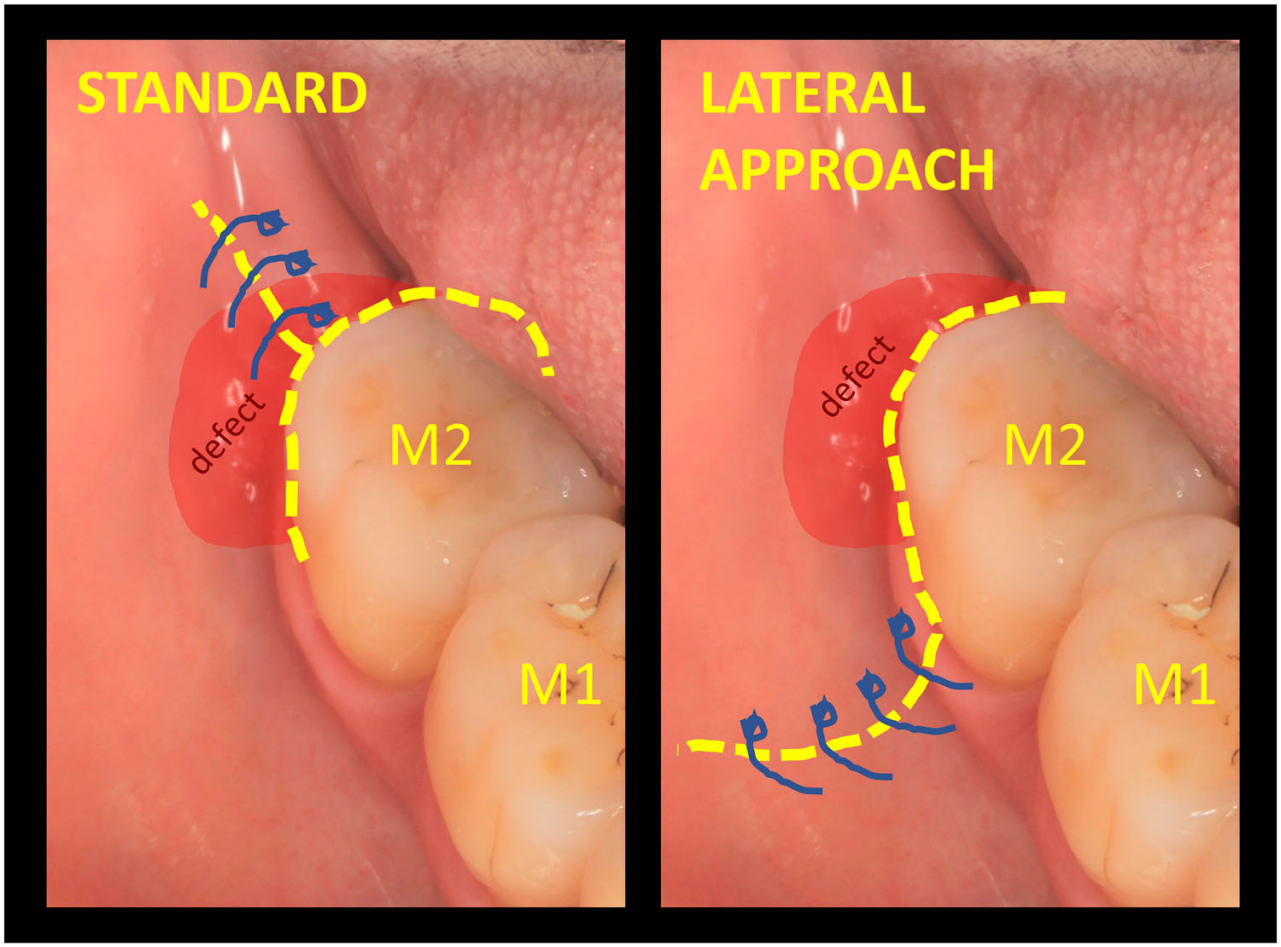
Comparison of incision lines and flap designs. Left – standard crestal approach; right – suggested “lateral approach for edentulous ridge.”

**Figure 2:**
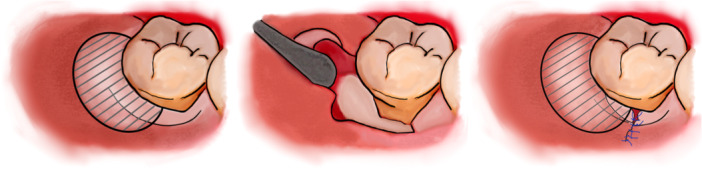
Surgical steps of the lateral approach for the regenerative treatment of intrabony periodontal defects associated with an edentulous ridge. Defects related to third molar removal generally extend from distal to mid‐buccal. Defects that are not related to third molar extraction present instead as distal defects.

We propose a novel surgical technique with a specific flap design, called the “lateral approach for edentulous ridge,” to prevent these complications and guarantee an undisturbed healing environment (see Figure 1 for comparison; see also Figure 2).

The purpose of this study was to describe and evaluate the “lateral approach” for the regenerative surgical treatment of intrabony periodontal defects associated with an edentulous ridge in a pilot case series using EMD and bone grafting with deproteinized bovine bone mineral. The goal of this method is to prevent the most common complications of the unchallenged gold‐standard flap design for this specific indication and to create more favorable conditions for periodontal regeneration.

## 2 Materials and Methods

The PROCESS (Preferred Reporting Of CasE Series in Surgery) guidelines for improving the quality of reports (Mathew et al. 2023) were followed in the preparation of the present manuscript.

### 2.1 Experimental Design

The study protocol was approved by the Ethics Committee of St. Anne's University Hospital, Brno, Czech Republic (approval no. 06 V/2023; project no. IIT/2023/05), and was carried out in full accordance with the Helsinki Declaration of 1975, as revised in 2000. Informed consent was obtained from all participants.

This study was designed as a single‐center prospective case series. Individuals presenting with at least one periodontal defect with a residual PPD ≥ 5 mm at the distal aspect of the lower second molar were screened consecutively for eligibility. Only defects with an intrabony component ≥ 4 mm, measured on digital periapical radiographs, and in the absence of a third molar, were included, regardless of their association with a third molar removal. Surgical treatment was performed only once the nonsurgical phase of periodontal therapy was completed, and a 3‐month healing period was allowed. Patients were considered eligible to participate in this study only if they had good systemic health, were nonsmokers, could maintain good oral hygiene (full‐mouth plaque score [FMPS] < 20%; full‐mouth bleeding score [FMBS] < 20%), and were willing to comply with the study protocol.

### 2.2 Study Sample

Seven systemically healthy nonsmokers (2 men, 5 women; median age: 42 years; IQR: 35–60 years) were enrolled in this case series. One defect per participant was treated in this study. None of the patients were lost to follow‐up. All included patients showed excellent levels of self‐performed plaque control, as represented by a median FMPS of 8% (IQR 4–16) and a median FMBS of 6% (IQR 4–18). All patients had a single intrabony defect distal to the lower second molar (five on the right and two on the left), with 1–3 remaining bony walls containing the lesion (three with 3 walls, three with 2 walls, and one with 1 wall). All included teeth were vital and presented with the maximum first‐degree furcation involvement without increased mobility or suppuration. None of the variables changed during the study period.

### 2.3 Surgery and Flap Design

All surgical procedures were performed at the St. Anne's University Hospital by the same experienced surgeon (F.H.) from February to June 2023.

**Figure 3:**
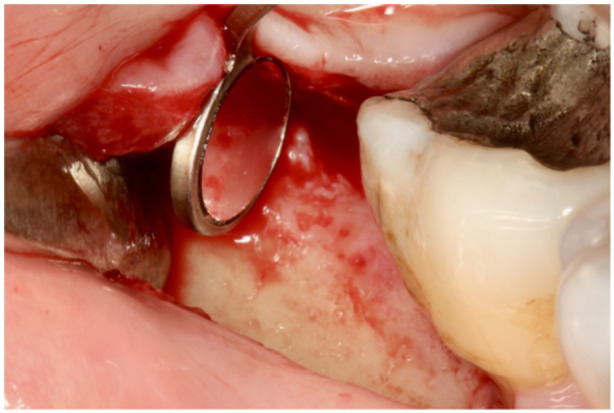
The distal wall of the treated tooth can be checked for plaque and calculus using a micro‐mirror.

**Figure 4:**
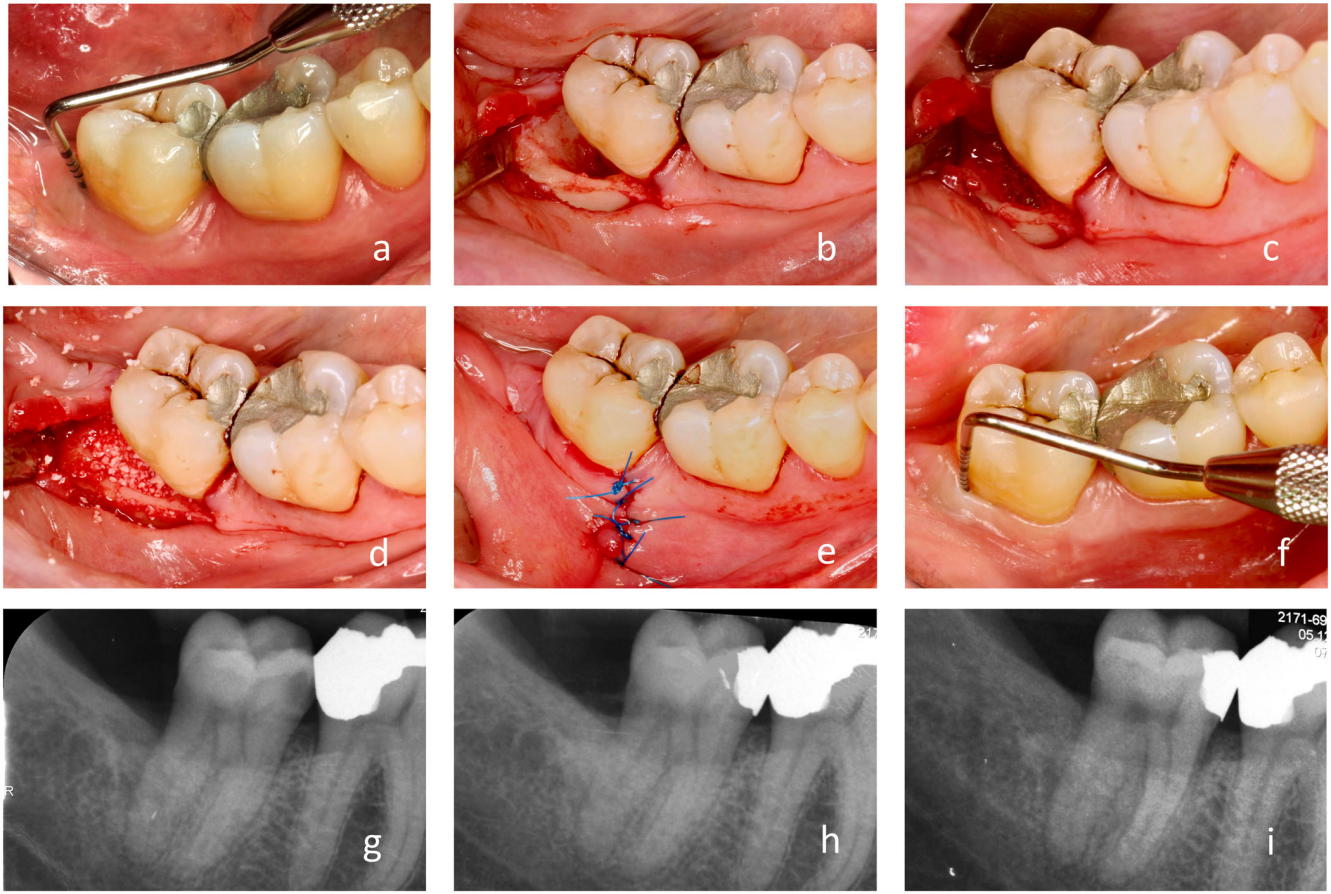
Representative case of regenerative treatment of intrabony periodontal defect associated with edentulous ridge with the suggested lateral approach. (a) Initial probing of 8 mm, a lesion distal to the lower right second molar associated with a complicated extraction of a fully erupted wisdom tooth 15 years ago. (b) Adequate view and access to the three‐walled defect obtained after elevation and hyper‐mobilization of the flap and granulation tissue removal. (c) Amelogenins applied for 4 min on the dry surface of the defect‐associated root after its scaling and root planing. (d) Bone substitute mixed with amelogenins used to fill up the defect. (e) Final suture clear of the actual defect, and also note the localization and shape of the initial vertical incision. (f) Probing 6 months after surgery. (g) Initial radiograph with a well‐defined intrabony defect with marked corticalization of its distal wall, extending close to the apex. (h) Immediate postsurgical radiograph with a bone substitute filling up the lesion. (i) 6 month radiograph with well‐integrated and partly remodeled bone substitute, radiopaque tissue occupying the lesion, and faded demarcation of the former defect.

The flap design for the lateral approach was defined as a curved vertical incision on the buccal side opposite the treated defect and a sulcular incision on the buccal and defect‐associated sides. A full‐thickness flap was raised and hyper‐mobilized, ensuring proper access while maintaining the uncut soft tissues above the defect. For flap elevation, both sharp and blunt dissections were performed using instruments such as a periosteal elevator, a micro‐blade, and/or tunneling instruments. After removing the granulation tissue, an adequate view of the distobuccal, distal, or even distolingual aspects of the treated molar was obtained using a micro‐mirror, and checked for possible calculus deposits (see Figure 3). The affected root surface was scaled using an ultrasound device (slim and furcation‐dedicated tips) and a Gracey curette. Subsequently, a regenerative strategy was applied. Amelogenins (Emdogain; Straumann, Basel, Switzerland) and bovine bone xenograft (BioOss; Geistlich, Wolhusen, Switzerland) were used for all the cases treated within this series. Amelogenins were applied to dry root surfaces for 4 min and suctioned. A mixture of bone graft and amelogenins was used to fill the defect, and a slight overfill was accepted. Re‐adaptation of the flap was passive. The vertical incision was closed with interrupted sutures (Resolon 5‐0; Resorba, Domažlice, Czechia). This procedure is illustrated in Figure 4.

### 2.4 Postsurgical Care

Prophylactic systemic antibiotics (amoxicillin/clavulanic acid 875 mg/125 mg twice daily for 7 days) were prescribed, and patients were given specific instructions for home care, which included rinsing with 0.2% chlorhexidine digluconate thrice daily for 2 weeks. Brushing of the affected area was not allowed for 3 weeks.

All patients were required to follow a strict postoperative schedule. Nine days after the surgery, the patients were checked, and their sutures were removed. The treated area was disinfected, and patients were given specific instructions for at‐home care. After 24 days, the wounds were checked again for possible late complications, and the patients were re‐instructed regarding their at‐home care. At 3 months, a dental hygienist conducted a check‐up to remove any deposits and reinforce self‐performed dental hygiene. Finally, a comprehensive check‐up, examination, and re‐evaluation were scheduled at 6 months.

### 2.5 Clinical Parameters

The primary objective of the present study was to evaluate the gain of clinical attachment level (CAL) at the distobuccal aspect of the lower second molar between baseline and 6 months post‐surgery. Reduction in PPD was considered the secondary treatment outcome.

Complementary periodontal parameters (bleeding on probing [BOP], suppuration, furcation involvement, mobility, and pulp sensibility) were recorded and analyzed before and 6 months after surgery. All periodontal measurements were performed using a University of North Carolina probe (HuFriedyGroup, Chicago, IL, USA) at six sites per tooth. During each surgery, the number of bony walls was determined and recorded, along with the operation duration.

Lastly, the study aimed to evaluate patient‐reported outcome measures (PROMs) throughout the study period: pain, swelling, need for analgesics, and discomfort during brushing. These parameters were recorded upon questionnaire at every visit following the surgery (9 days, 24 days, 3 months, 6 months).

### 2.6 Radiographic Evaluation

Digital periapical radiographs were obtained using the paralleling technique with a Rinn holder and a beam‐guiding system at three time points: baseline (initial), immediately after surgery to visualize the grafting material (and eventual overfill), and 6 months post‐surgery. To maximize the comparability of the X‐ray series, minor changes in the projection geometry were made through post‐acquisition image processing, and a perfect overlap of images within each series was achieved. The image intensities were normalized based on the grayscale values of the surrounding air, dentin, and enamel of the examined teeth (Huang et al. 2022; Jeffcoat et al. 1996).

### 2.7 Statistical Methods

Statistical analysis was performed using the *R* programming language (R Foundation for Statistical Computing, Vienna, Austria) in the integrated development environment *R studio*. Data were tested for normality using the Shapiro–Wilk test and graphically using Q–Q plots and histograms. The paired Wilcoxon test was used to compare variables at two time points. Continuous variables were presented as medians and interquartile ranges (IQRs). Results with a *p*‐value < 0.05 were considered statistically significant.

## 3 Results

### 3.1 Clinical Outcomes

All defects were treated using the lateral surgical approach, as described previously, with a median operation duration of 64 min (IQR 55–67 min). No patients experienced any relevant postoperative complications (severe pain, infection, bleeding, graft leakage, or flap dehiscence). Optimal plaque control levels were maintained throughout the study (FMPS < 20%, FMBS < 20%), and all patients adhered to the follow‐up protocol. None of the patients reported pain, swelling, need for analgesics, or discomfort while brushing after > 9 days post‐surgery.

**Figure 5:**
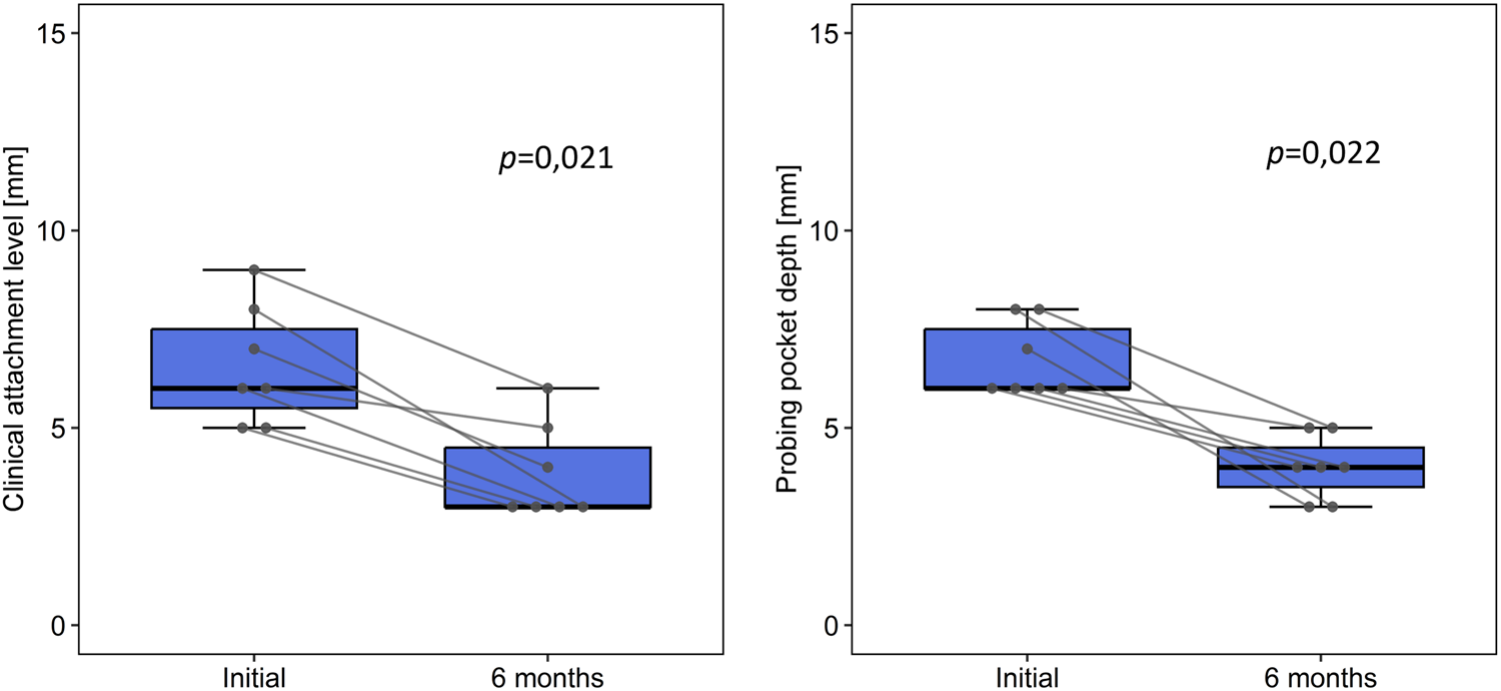
Difference of CAL and PPD between the initial and 6‐month evaluations. Expressed with respective *p*‐values, paired Wilcoxon test.

Residual probing depth of ≤ 5 mm was accomplished in all of the cases, which is considered an acceptable outcome of therapy (Chapple et al. 2018). Primary wound healing was observed in 100% of cases. At 6‐month re‐evaluation, the initial median CAL of 6 mm (IQR 5–8 mm) was reduced to 3 mm (3–5 mm). The corresponding median PPD decreased from 6 mm (IQR 6–8 mm) to 4 mm (IQR 3–5 mm). Both CAL and PPD changes were considered statistically significant (*p* < 0.05). The results are shown in Figure 5.

The treatment had an unambiguously positive effect on both CAL and PPD. Clinical attachment gain and PPD reduction were observed in all the cases. A significant positive effect was observed in the distobuccal aspect, which represented the deepest part of the defect. The distolingual CAL and PPD measurements also showed positive but insignificant changes (*p* > 0.05). The treatment did not affect other sites and parameters (tooth mobility, furcation involvement, and BOP). The deeper the initial probing, the greater the changes in CAL and PPD. The number of bony walls in the defect did not significantly affect the outcomes (*p* > 0.05).

**Figure 6:**
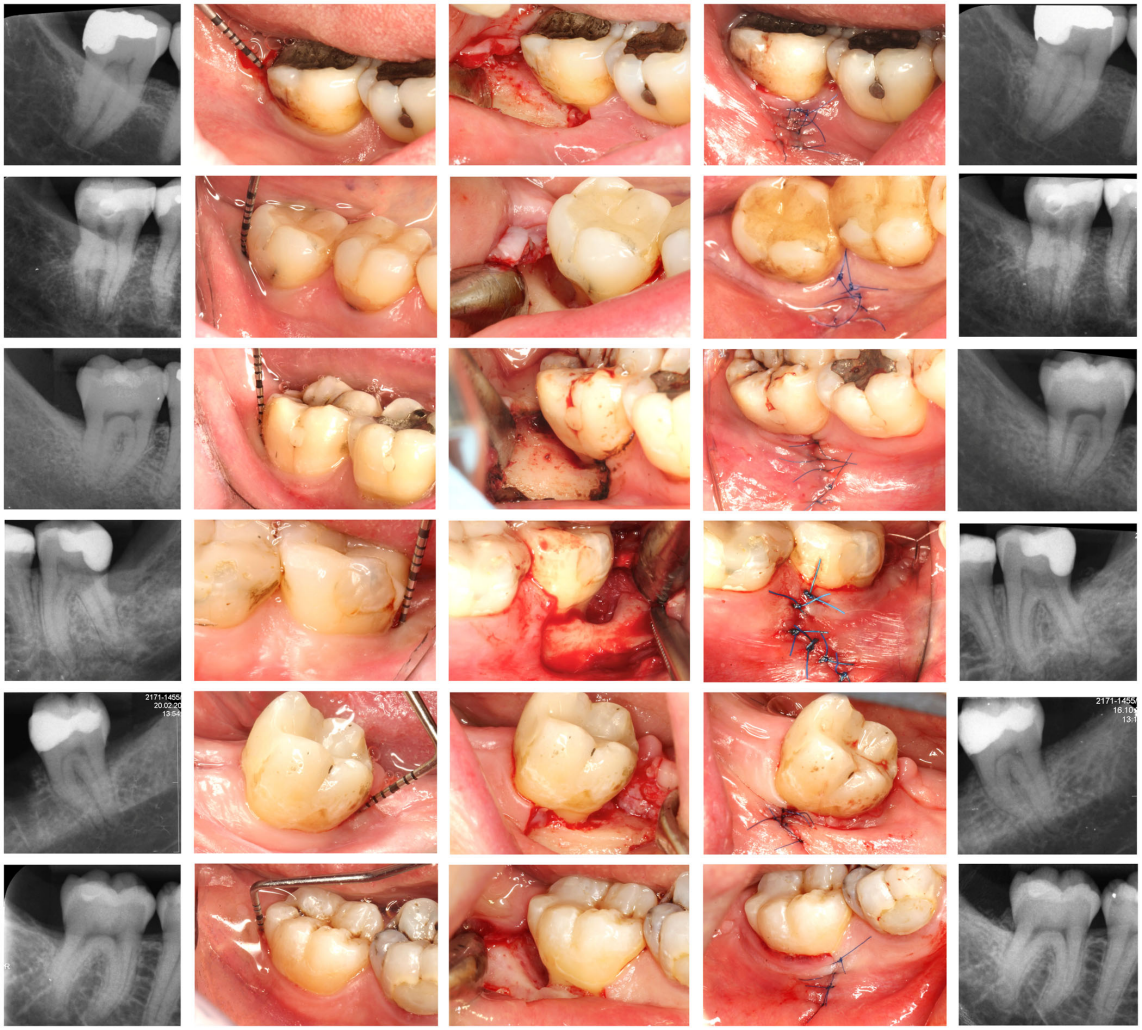
Overview of all the remaining cases included in the presented report. From left to right: initial radiograph; initial clinical situation; access to the defect after degranulation and scaling; final suture – note how far the suture is from the actual defect; and 6‐month radiograph.

Radiographic evaluation showed bone‐fill retention and partial remodeling of the grafting material 6 months post‐surgery in all treated cases (Figure 6).

## 4 Discussion

This report aimed to describe and evaluate the lateral approach for the regenerative surgical treatment of intrabony periodontal defects associated with the edentulous alveolar ridge, which also occur frequently in association with the removal of the third molar. This is the first technique specifically designed for these neglected defects, in contrast to the various approaches for papilla‐associated lesions (Cortellini and Tonetti 2011; Aslan et al. 2020; Nibali et al. 2019).

Defects at the distal aspect of the lower second molars were chosen as suitable study models. Such defects represent intrabony lesions next to the edentulous ridge, and present a challenging clinical situation. They are also often resistant to nonsurgical therapeutic efforts. They usually occur in conjunction with the removal of third molars, particularly when they are impacted mesially or horizontally (Singh et al. 2022; Kan et al. 2002). This is due to their relationship with the periodontium of the second molar (Yang et al. 2023). The development of a post‐extraction periodontal defect on the distal aspect of the second molar is presumed if there was a pre‐extraction probing pocket depth (PPD) of ≥ 7 mm and a history of periodontitis (Kugelberg et al. 1991; Passarelli et al. 2019). Analysis of this association was not within the scope of this trial.

In this prospective case series, clinical efficiency of the approach was shown, as evidenced by a statistically significant positive change in both CAL and PPD, and primary healing. In all cases, the general clinical objective of periodontal therapy (PPD ≤ 5 mm) (Chapple et al. 2018) was met, maintainable sulci were created, and good self‐performed plaque control levels ensure stable results (Matuliene et al. 2008). Comparable results were obtained in a previously reported pilot case, which was not included in the present case series (Hromčík and Halusková 2023).

The lateral approach, similar to other “conservative” or papilla‐preserving approaches in periodontal surgery, shares the common aim of creating a clinical environment in which the delicate biological process of regeneration occurs in a space where a very stable flap provides the room and stability of the clot and its biological protection. This was secured by only one vertical incision away from the defect, elevation of only the buccal flap, and preservation of the intact soft tissues above the defect. This resulted in primary healing, flap integrity, and no dehiscence, graft exposure, or infectious complications in all our cases. Studies using other “conservative” techniques have confirmed that intact soft tissues covering the defect led to comparable results with or even without the use of biomaterials (Cortellini and Tonetti 2011; Trombelli et al. 2010).

During the 6‐month observation period, this study successfully demonstrated reduction of PPD to ≤ 5 mm in 100% cases and achieved the overall clinical objective of periodontal therapy. The rather short study period of 6 months was sufficient to produce relevant data and show clear CAL gain and PPD reduction, which is likely to become even more evident with time. A 6‐month observation period is typically considered the minimum interval for evaluating periodontal healing after regenerative treatment (Carvalho Dutra et al. 2019; Saito et al. 2019).

The radiographic evaluation documents partial remodeling of bone graft and residual intrabony defects (Figure 6). The effect of grafting was more evident in the deeper lesions. Defects related to third molar extraction generally extend from the distal to the mid‐buccal aspect of the second molar. Micro‐CT imaging may also be appropriate for assessing bone healing in the buccal part of the lesion. Intraoral radiographs were analyzed to illustrate the bone changes in the distal part of the defects. Drawing conclusions regarding bone regeneration or gain was beyond the scope of this study. However, the specific flap design of the presented lateral surgical approach seems to be responsible for the improved graft and wound stability.

The goal of the technique is not only to reduce probing and create more favorable conditions for healing but also to prevent the most common complications of the gold‐standard flap design for this indication (Figure 1). These are mostly related to the incision immediately above the defect and use of membranes, leading to flap dehiscence and membrane exposure in 50%–100% of cases (Machtei 2001; Cortellini et al. 1990; Sanz et al. 2004). In this case series, no complications have been observed at all (as shown in Figures 2, 3, and 6); suturing was performed only where the former buccal vertical incision was away from the actual defect and distant from the regenerative materials. Therefore, even in cases of suture failure and marginal flap dehiscence, the defect itself would not be affected, materials would not be exposed or infected, and regeneration would not be jeopardized.

In contrast to the gold‐standard technique defined by the crestal approach, as described in an epoch‐making clinical guideline by Cortellini and Tonetti (2015), the lateral approach does not require the use of a membrane. The intact flap above the defect serves as an additional stable “wall,” confers stability to the blood clot underneath, supports undisturbed healing, and provides membrane‐like protection of the grafting material. The lateral approach appears to be less costly, easier to perform, and less prone to complications. Similar to the lateral approach for papilla‐associated defects, described as “entire papilla preservation flap” by Aslan et al. (2017), healing in this case series occurred with no complications and by primary intention in all of the cases. Within the limitations of this study, the presented lateral approach was shown to be safe and predictable, with a reasonable operation duration, low levels of morbidity, and consistent results.

The lateral surgical approach is suitable for intrabony defects with 1–3 bony walls, which generally have good regenerative potential (Cortellini and Tonetti 2011), preferably with disto‐buccal extension. Suprabony residual defects associated with the edentulous ridge should preferably be treated with access flaps or resective periodontal surgery (Graziani et al. 2018). In cases of a rather lingual extension of the treated intrabony defect, the lateral approach would not be feasible, and a crestal approach would be required instead. Buccal placement of the crestal incision should then be preferred, depending on the extent of the actual defect.

Based on clinical experience, the lateral approach is better and easier to perform if there is at least 1 cm of the horizontal bony crest at the ramus mandibulae distal to the second molar and a minimal amount of 2 mm of keratinized gingiva surrounding the treated tooth. Access to the defect may be adequate in all cases with the use of a curved vertical incision extending 5–10 mm past the MG junction, abundant distal mobilization of the flap, and a micro‐mirror.

The limitations of the present study included the lack of a control group, a minimal acceptable observation period, and a relatively small sample size. The sample size was determined according to the anticipated CAL gain and adjusted to match the ethical criteria for testing a previously undescribed surgical method.

The main strengths of this study are its focus on previously neglected defects, innovative flap design for edentulous ridge, its prospective design, which included a systematic evaluation of very similar defects treated using the same uniform approach by the same operator, and a strict follow‐up protocol.

Future studies may further assess our findings, compare the outcomes of the lateral approach to other techniques, and extrapolate the principles of the lateral approach to localizations unrelated to the second lower molars. Future studies should also evaluate whether the technique is relevant for both mesial and distal defects in both frontal and distal areas, and whether the use of biomaterials is necessary for clinical success.

## 5 Conclusions

Within the limitations of this prospective case series, the following conclusions were drawn:

- The lateral approach is a valid technique for regenerative periodontal surgery, specifically tailored for the regenerative treatment of intrabony periodontal defects associated with an edentulous ridge, achieving the goals of clinical attachment gain, PPD reduction, bone grafting, and enhancement of periodontal regeneration with little predisposition to failure.
- Common complications of a gold‐standard flap design can be prevented by shifting the most vulnerable area away from the regenerated site. The intact soft tissue above the defect protects the wound and keeps the regenerative material undisturbed during healing.
- The procedure is not time‐consuming or difficult to perform.

We suggest using this approach especially in distobuccal intrabony defects in the second molars, which frequently develop after third molar removal. Similarly, it may also be suitable for regenerating any intrabony lesions associated with an edentulous ridge. Further studies are required to validate these findings.

## Author Contributions

**Filip Hromčík:** conceptualization, data curation (equal), funding acquisition, investigation (equal), methodology (lead), visualization, and writing – original draft preparation. **Adéla Halusková:** data curation (equal), investigation (equal), methodology (supporting), and writing – review and editing (supporting). **Lydie Izakovičová Hollá:** supervision and writing – review and editing (lead).

## Ethics Statement

The study protocol was approved by the Ethics Committee of St. Anne's University Hospital, Brno, Czech Republic (approval no. 06 V/2023; project no. IIT/2023/05), and was in full accordance with the Helsinki Declaration of 1975, as revised in 2000.

## Consent

Informed consent was obtained from all participants.

## Conflicts of Interest

The authors declare no conflicts of interest.

## Supporting information

Supporting information.

## Data Availability

The data that support the findings of this study are available from the corresponding author upon reasonable request. All the data are available on request from the authors.
